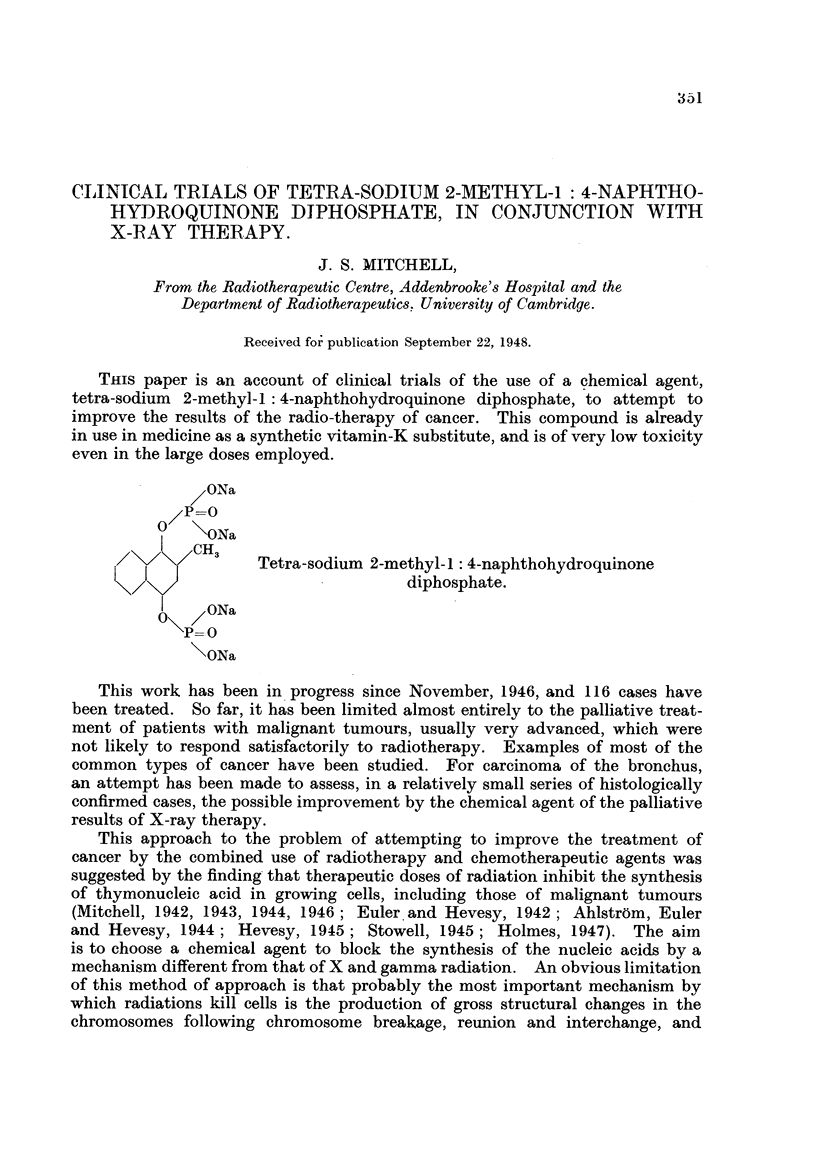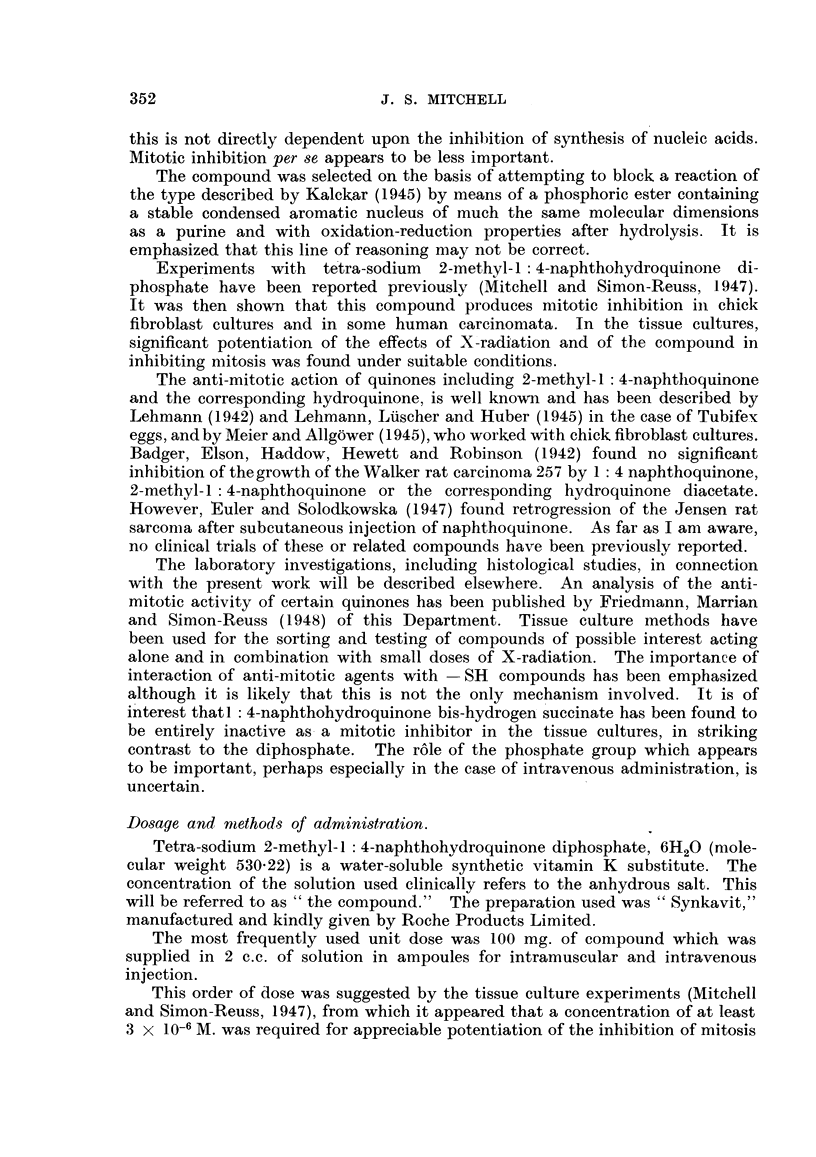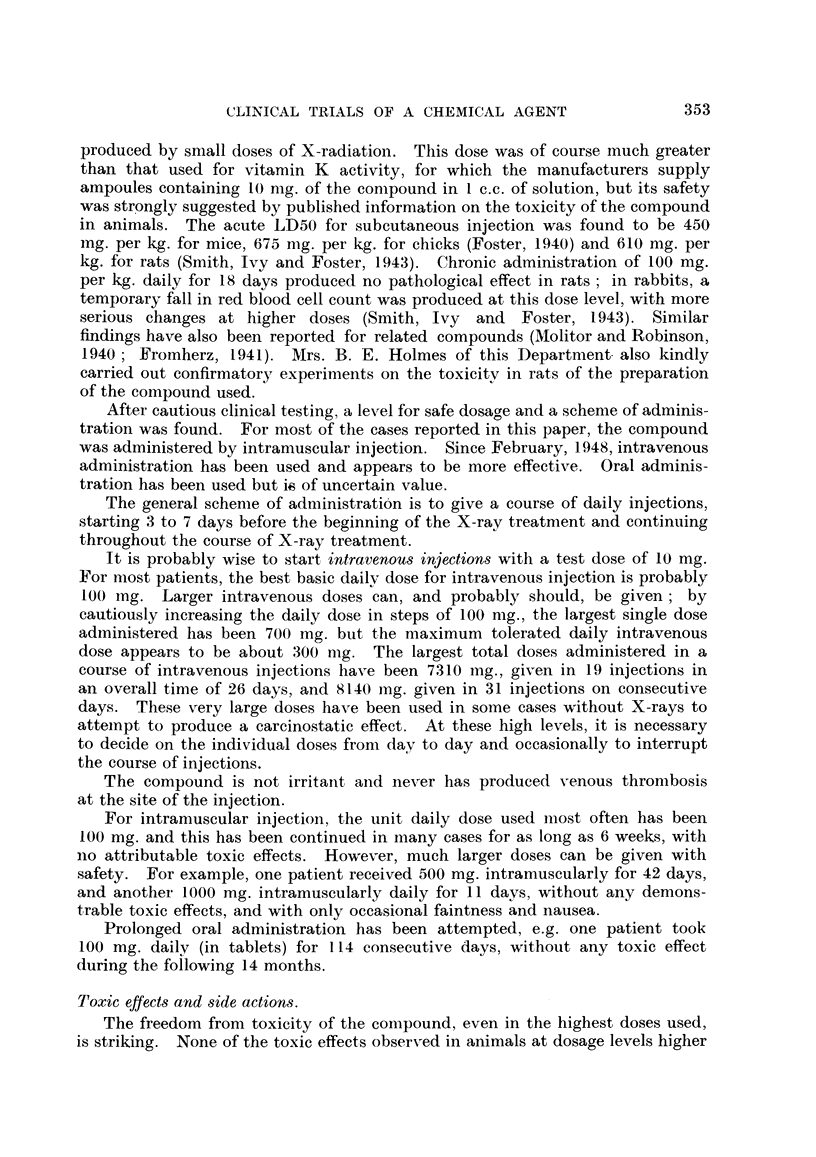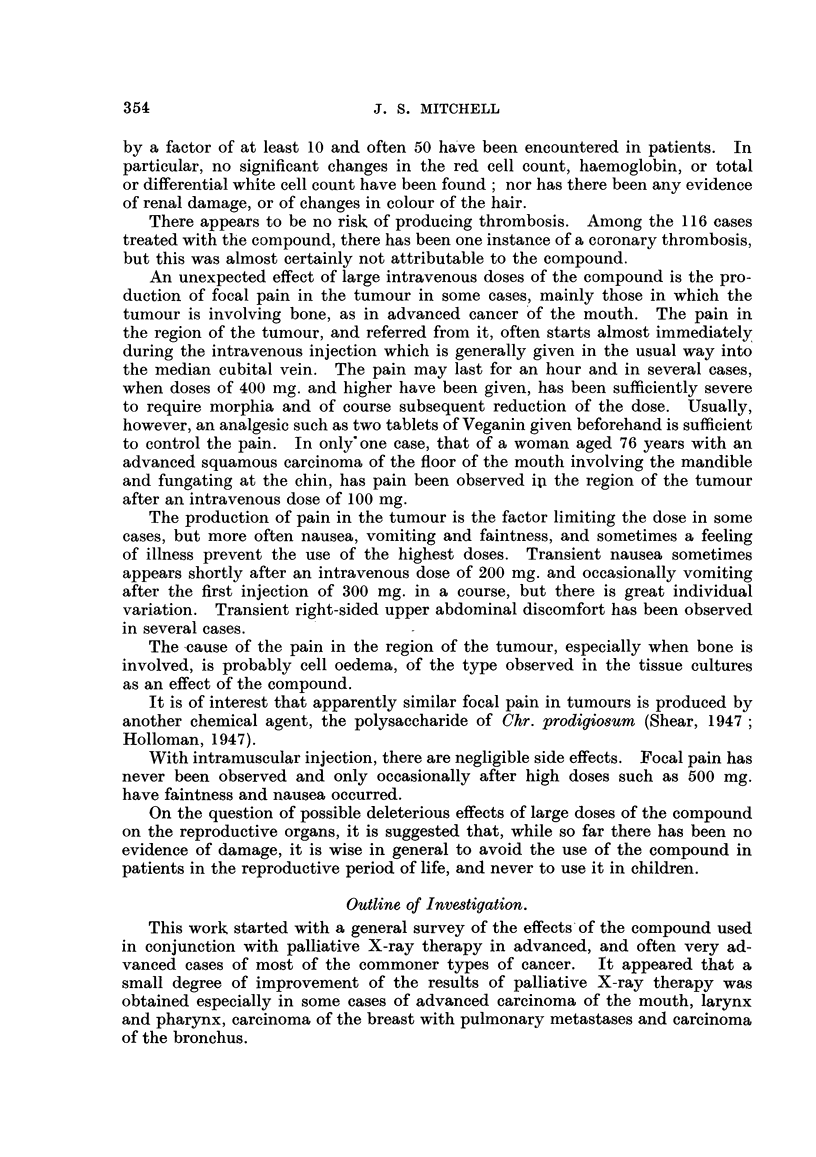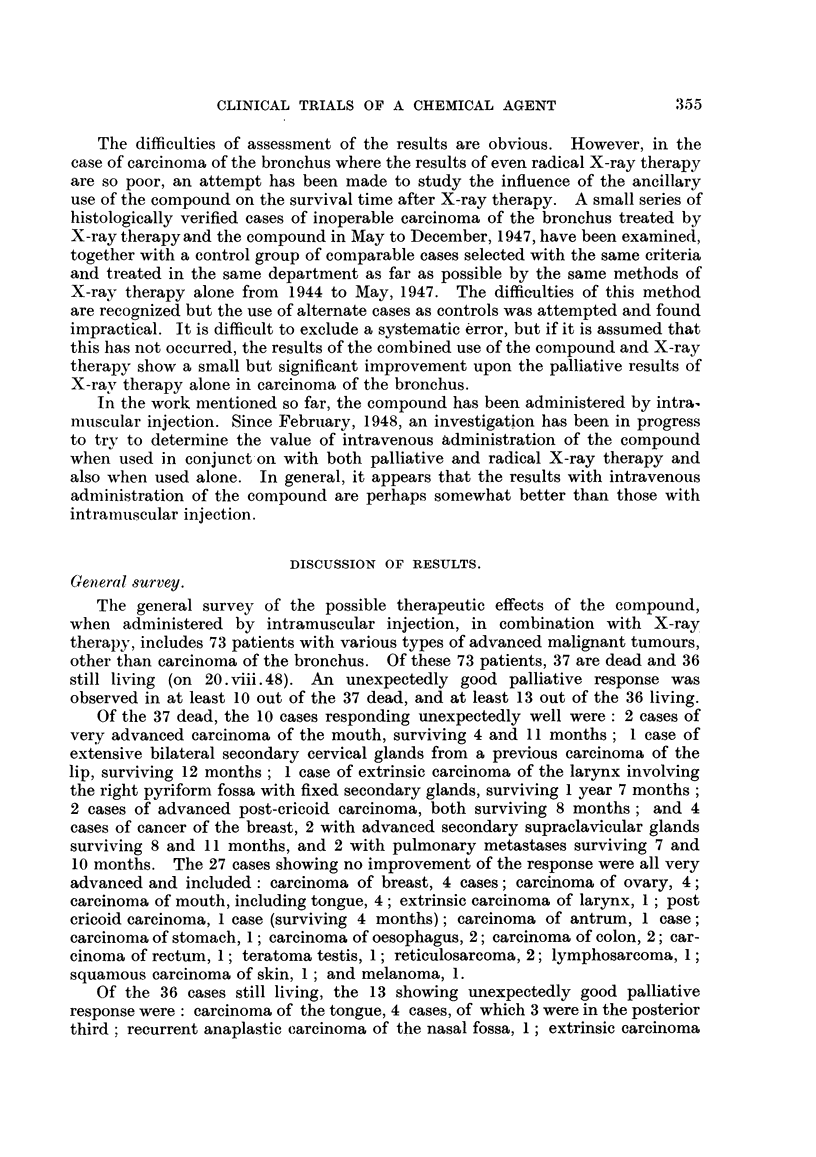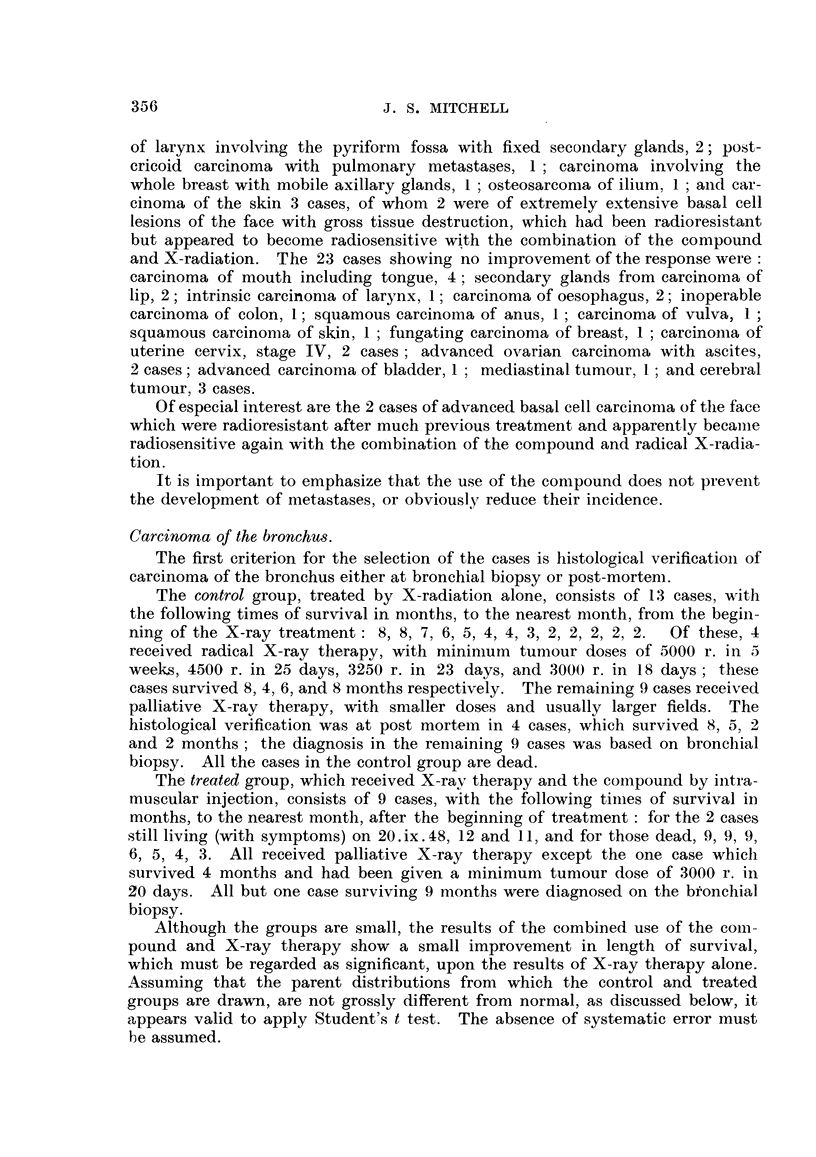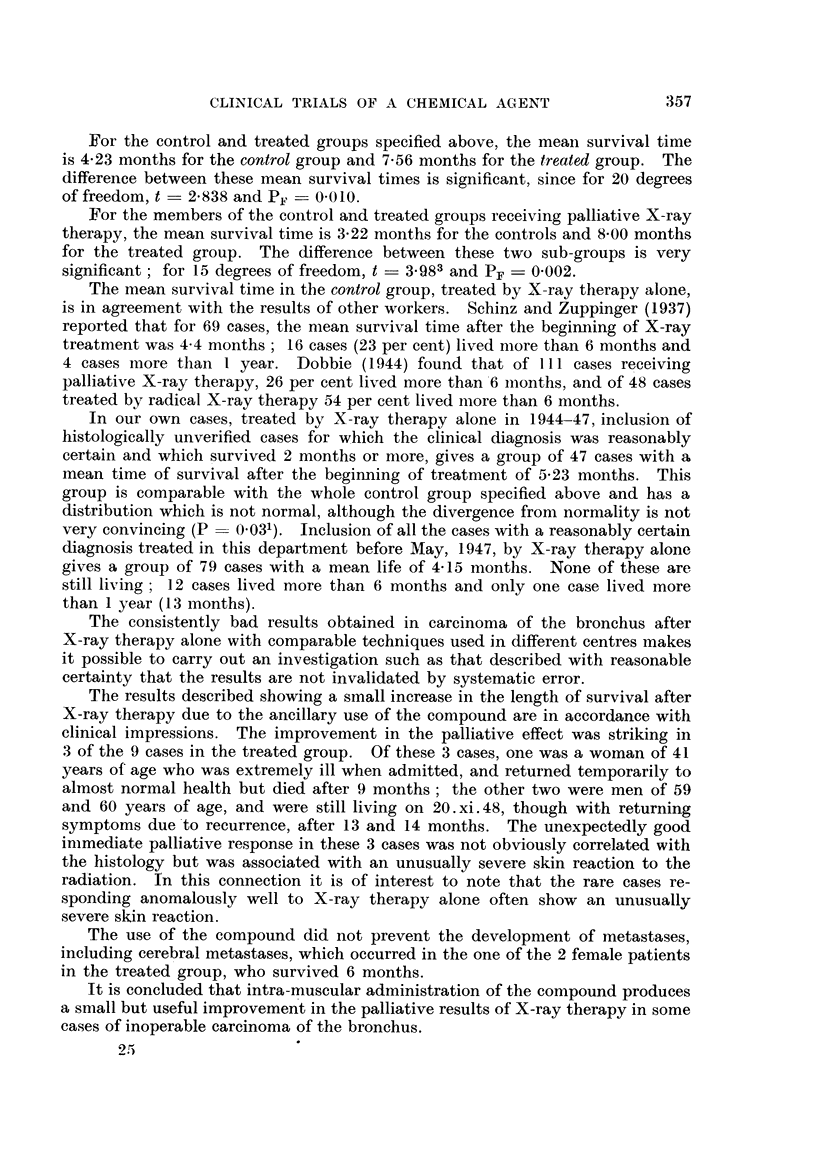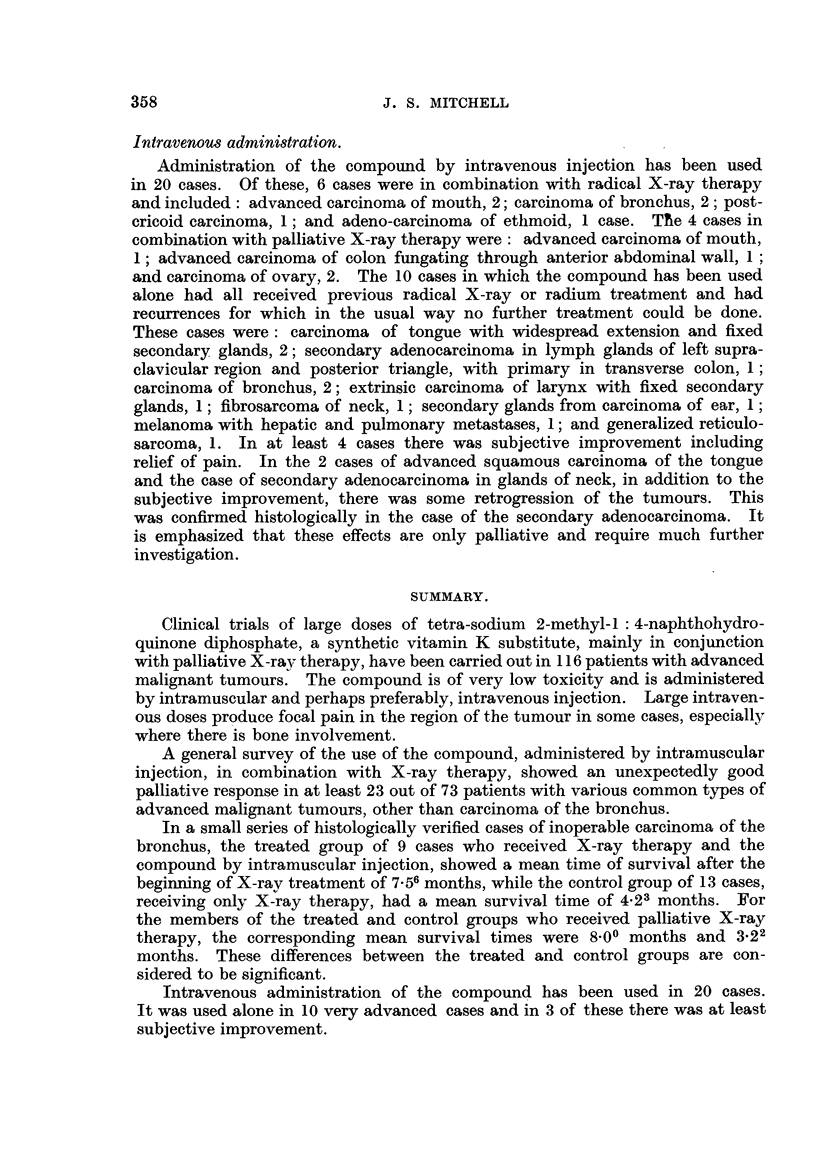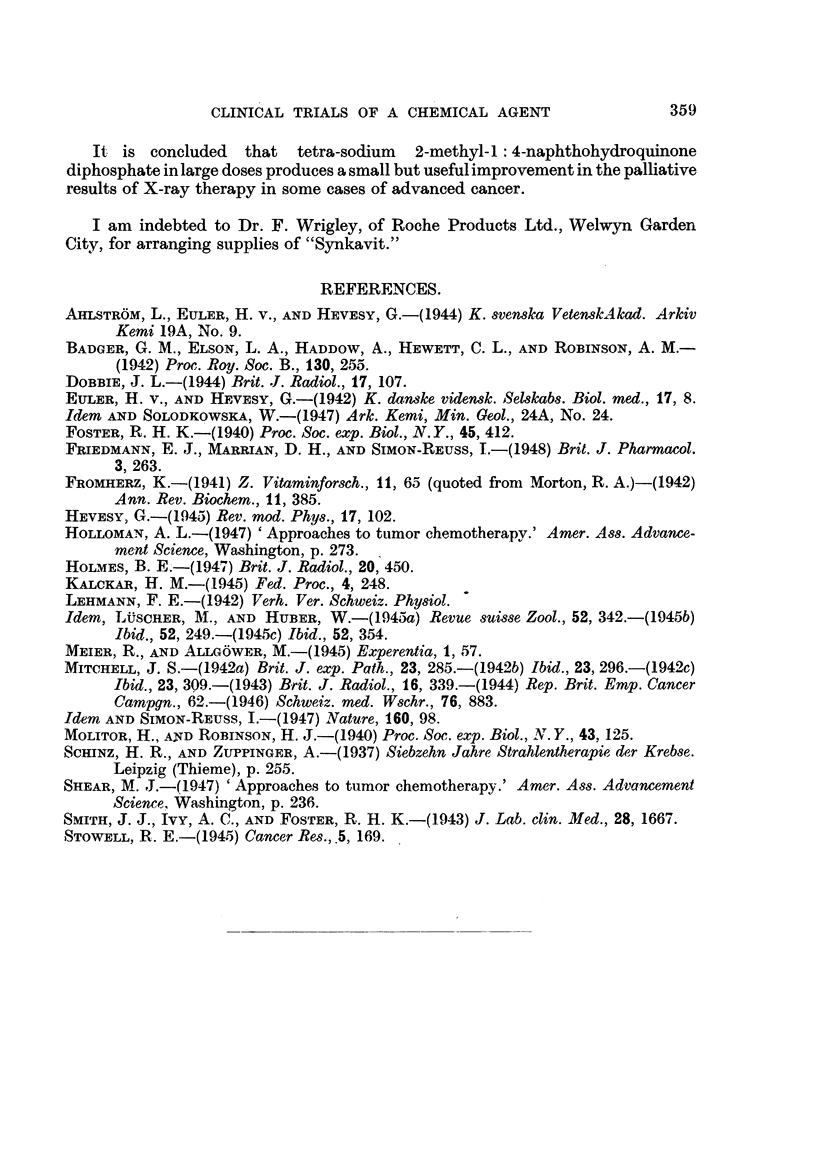# Clinical Trials of Tetra-Sodium 2-Methyl-1:4-Naphthohydroquinone Diphosphate, in Conjunction with X-ray Therapy

**DOI:** 10.1038/bjc.1948.38

**Published:** 1948-12

**Authors:** J. S. Mitchell


### CLINICAL TRIALS OF TETRA-SODIUM 2-METHYL-1 : 4-NAPHTHOHYDROQUINONE DIPHOSPHATE, IN CONJUNCTION WITH X-RAY THERAPY.

J. S. MITCHELL,

*From the Radiotherapeutic Centre, Addenbrooke's Hospital and the Department of Radiotherapeutics, University of Cambridge.*

Received for publication September 22, 1948.

THIS paper is an account of clinical trials of the use of a chemical agent, tetra-sodium 2-methyl-1 : 4-naphthohydroquinone diphosphate, to attempt to improve the results of the radio-therapy of cancer. This compound is already in use in medicine as a synthetic vitamin-K substitute, and is of very low toxicity even in the large doses employed.

Tetra-sodium 2-methyl-1 : 4-naphthohydroquinone diphosphate.

This work has been in progress since November, 1946, and 116 cases have been treated. So far, it has been limited almost entirely to the palliative treatment of patients with malignant tumours, usually very advanced, which were not likely to respond satisfactorily to radiotherapy. Examples of most of the common types of cancer have been studied. For carcinoma of the bronchus, an attempt has been made to assess, in a relatively small series of histologically confirmed cases, the possible improvement by the chemical agent of the palliative results of X-ray therapy.

This approach to the problem of attempting to improve the treatment of cancer by the combined use of radiotherapy and chemotherapeutic agents was suggested by the finding that therapeutic doses of radiation inhibit the synthesis of thymonucleic acid in growing cells, including those of malignant tumours (Mitchell, 1942, 1943, 1944, 1946 ; Euler and Hevesy, 1942 ; Ahlström, Euler and Hevesy, 1944 ; Hevesy, 1945 ; Stowell, 1945 ; Holmes, 1947). The aim is to choose a chemical agent to block the synthesis of the nucleic acids by a mechanism different from that of X and gamma radiation. An obvious limitation of this method of approach is that probably the most important mechanism by which radiations kill cells is the production of gross structural changes in the chromosomes following chromosome breakage, reunion and interchange, and this is not directly dependent upon the inhibition of synthesis of nucleic acids. Mitotic inhibition *per se* appears to be less important.

The compound was selected on the basis of attempting to block a reaction of the type described by Kalckar (1945) by means of a phosphoric ester containing a stable condensed aromatic nucleus of much the same molecular dimensions as a purine and with oxidation-reduction properties after hydrolysis. It is emphasized that this line of reasoning may not be correct.

Experiments with tetra-sodium 2-methyl-1 : 4-naphthohydroquinone diphosphate have been reported previously (Mitchell and Simon-Reuss, 1947). It was then shown that this compound produces mitotic inhibition in chick fibroblast cultures and in some human carcinomata. In the tissue cultures, significant potentiation of the effects of X-radiation and of the compound in inhibiting mitosis was found under suitable conditions.

The anti-mitotic action of quinones including 2-methyl-1 : 4-naphthoquinone and the corresponding hydroquinone, is well known and has been described by Lehmann (1942) and Lehmann, Lüscher and Huber (1945) in the case of Tubifex eggs, and by Meier and Allgöwer (1945), who worked with chick fibroblast cultures. Badger, Elson, Haddow, Hewett and Robinson (1942) found no significant inhibition of the growth of the Walker rat carcinoma 257 by 1 : 4 naphthoquinone, 2-methyl-1 : 4-naphthoquinone or the corresponding hydroquinone diacetate. However, Euler and Solodkowska (1947) found retrogression of the Jensen rat sarcoma after subcutaneous injection of naphthoquinone. As far as I am aware, no clinical trials of these or related compounds have been previously reported.

The laboratory investigations, including histological studies, in connection with the present work will be described elsewhere. An analysis of the anti-mitotic activity of certain quinones has been published by Friedmann, Marrian and Simon-Reuss (1948) of this Department. Tissue culture methods have been used for the sorting and testing of compounds of possible interest acting alone and in combination with small doses of X-radiation. The importance of interaction of anti-mitotic agents with —SH compounds has been emphasized although it is likely that this is not the only mechanism involved. It is of interest that 1 : 4-naphthohydroquinone bis-hydrogen succinate has been found to be entirely inactive as a mitotic inhibitor in the tissue cultures, in striking contrast to the diphosphate. The rôle of the phosphate group which appears to be important, perhaps especially in the case of intravenous administration, is uncertain.

*Dosage and methods of administration.*

Tetra-sodium 2-methyl-1 : 4-naphthohydroquinone diphosphate, $6H_2O$ (molecular weight 530·22) is a water-soluble synthetic vitamin K substitute. The concentration of the solution used clinically refers to the anhydrous salt. This will be referred to as " the compound." The preparation used was " Synkavit," manufactured and kindly given by Roche Products Limited.

The most frequently used unit dose was 100 mg. of compound which was supplied in 2 c.c. of solution in ampoules for intramuscular and intravenous injection.

This order of dose was suggested by the tissue culture experiments (Mitchell and Simon-Reuss, 1947), from which it appeared that a concentration of at least $3 \times 10^{-6}$ M. was required for appreciable potentiation of the inhibition of mitosis produced by small doses of X-radiation. This dose was of course much greater than that used for vitamin K activity, for which the manufacturers supply ampoules containing 10 mg. of the compound in 1 c.c. of solution, but its safety was strongly suggested by published information on the toxicity of the compound in animals. The acute LD50 for subcutaneous injection was found to be 450 mg. per kg. for mice, 675 mg. per kg. for chicks (Foster, 1940) and 610 mg. per kg. for rats (Smith, Ivy and Foster, 1943). Chronic administration of 100 mg. per kg. daily for 18 days produced no pathological effect in rats ; in rabbits, a temporary fall in red blood cell count was produced at this dose level, with more serious changes at higher doses (Smith, Ivy and Foster, 1943). Similar findings have also been reported for related compounds (Molitor and Robinson, 1940 ; Fromherz, 1941). Mrs. B. E. Holmes of this Department also kindly carried out confirmatory experiments on the toxicity in rats of the preparation of the compound used.

After cautious clinical testing, a level for safe dosage and a scheme of administration was found. For most of the cases reported in this paper, the compound was administered by intramuscular injection. Since February, 1948, intravenous administration has been used and appears to be more effective. Oral administration has been used but is of uncertain value.

The general scheme of administration is to give a course of daily injections, starting 3 to 7 days before the beginning of the X-ray treatment and continuing throughout the course of X-ray treatment.

It is probably wise to start *intravenous injections* with a test dose of 10 mg. For most patients, the best basic daily dose for intravenous injection is probably 100 mg. Larger intravenous doses can, and probably should, be given ; by cautiously increasing the daily dose in steps of 100 mg., the largest single dose administered has been 700 mg. but the maximum tolerated daily intravenous dose appears to be about 300 mg. The largest total doses administered in a course of intravenous injections have been 7310 mg., given in 19 injections in an overall time of 26 days, and 8140 mg. given in 31 injections on consecutive days. These very large doses have been used in some cases without X-rays to attempt to produce a carcinostatic effect. At these high levels, it is necessary to decide on the individual doses from day to day and occasionally to interrupt the course of injections.

The compound is not irritant and never has produced venous thrombosis at the site of the injection.

For intramuscular injection, the unit daily dose used most often has been 100 mg. and this has been continued in many cases for as long as 6 weeks, with no attributable toxic effects. However, much larger doses can be given with safety. For example, one patient received 500 mg. intramuscularly for 42 days, and another 1000 mg. intramuscularly daily for 11 days, without any demonstrable toxic effects, and with only occasional faintness and nausea.

Prolonged oral administration has been attempted, e.g. one patient took 100 mg. daily (in tablets) for 114 consecutive days, without any toxic effect during the following 14 months.

*Toxic effects and side actions.*

The freedom from toxicity of the compound, even in the highest doses used, is striking. None of the toxic effects observed in animals at dosage levels higher by a factor of at least 10 and often 50 have been encountered in patients. In particular, no significant changes in the red cell count, haemoglobin, or total or differential white cell count have been found ; nor has there been any evidence of renal damage, or of changes in colour of the hair.

There appears to be no risk of producing thrombosis. Among the 116 cases treated with the compound, there has been one instance of a coronary thrombosis, but this was almost certainly not attributable to the compound.

An unexpected effect of large intravenous doses of the compound is the production of focal pain in the tumour in some cases, mainly those in which the tumour is involving bone, as in advanced cancer of the mouth. The pain in the region of the tumour, and referred from it, often starts almost immediately during the intravenous injection which is generally given in the usual way into the median cubital vein. The pain may last for an hour and in several cases, when doses of 400 mg. and higher have been given, has been sufficiently severe to require morphia and of course subsequent reduction of the dose. Usually, however, an analgesic such as two tablets of Veganin given beforehand is sufficient to control the pain. In only one case, that of a woman aged 76 years with an advanced squamous carcinoma of the floor of the mouth involving the mandible and fungating at the chin, has pain been observed in the region of the tumour after an intravenous dose of 100 mg.

The production of pain in the tumour is the factor limiting the dose in some cases, but more often nausea, vomiting and faintness, and sometimes a feeling of illness prevent the use of the highest doses. Transient nausea sometimes appears shortly after an intravenous dose of 200 mg. and occasionally vomiting after the first injection of 300 mg. in a course, but there is great individual variation. Transient right-sided upper abdominal discomfort has been observed in several cases.

The cause of the pain in the region of the tumour, especially when bone is involved, is probably cell oedema, of the type observed in the tissue cultures as an effect of the compound.

It is of interest that apparently similar focal pain in tumours is produced by another chemical agent, the polysaccharide of *Chr. prodigiosum* (Shear, 1947 ; Holloman, 1947).

With intramuscular injection, there are negligible side effects. Focal pain has never been observed and only occasionally after high doses such as 500 mg. have faintness and nausea occurred.

On the question of possible deleterious effects of large doses of the compound on the reproductive organs, it is suggested that, while so far there has been no evidence of damage, it is wise in general to avoid the use of the compound in patients in the reproductive period of life, and never to use it in children.

### *Outline of Investigation.*

This work started with a general survey of the effects of the compound used in conjunction with palliative X-ray therapy in advanced, and often very advanced cases of most of the commoner types of cancer. It appeared that a small degree of improvement of the results of palliative X-ray therapy was obtained especially in some cases of advanced carcinoma of the mouth, larynx and pharynx, carcinoma of the breast with pulmonary metastases and carcinoma of the bronchus.

The difficulties of assessment of the results are obvious. However, in the case of carcinoma of the bronchus where the results of even radical X-ray therapy are so poor, an attempt has been made to study the influence of the ancillary use of the compound on the survival time after X-ray therapy. A small series of histologically verified cases of inoperable carcinoma of the bronchus treated by X-ray therapy and the compound in May to December, 1947, have been examined, together with a control group of comparable cases selected with the same criteria and treated in the same department as far as possible by the same methods of X-ray therapy alone from 1944 to May, 1947. The difficulties of this method are recognized but the use of alternate cases as controls was attempted and found impractical. It is difficult to exclude a systematic error, but if it is assumed that this has not occurred, the results of the combined use of the compound and X-ray therapy show a small but significant improvement upon the palliative results of X-ray therapy alone in carcinoma of the bronchus.

In the work mentioned so far, the compound has been administered by intramuscular injection. Since February, 1948, an investigation has been in progress to try to determine the value of intravenous administration of the compound when used in conjunct on with both palliative and radical X-ray therapy and also when used alone. In general, it appears that the results with intravenous administration of the compound are perhaps somewhat better than those with intramuscular injection.

### DISCUSSION OF RESULTS.

*General survey.*

The general survey of the possible therapeutic effects of the compound, when administered by intramuscular injection, in combination with X-ray therapy, includes 73 patients with various types of advanced malignant tumours, other than carcinoma of the bronchus. Of these 73 patients, 37 are dead and 36 still living (on 20.viii.48). An unexpectedly good palliative response was observed in at least 10 out of the 37 dead, and at least 13 out of the 36 living.

Of the 37 dead, the 10 cases responding unexpectedly well were : 2 cases of very advanced carcinoma of the mouth, surviving 4 and 11 months ; 1 case of extensive bilateral secondary cervical glands from a previous carcinoma of the lip, surviving 12 months ; 1 case of extrinsic carcinoma of the larynx involving the right pyriform fossa with fixed secondary glands, surviving 1 year 7 months ; 2 cases of advanced post-cricoid carcinoma, both surviving 8 months ; and 4 cases of cancer of the breast, 2 with advanced secondary supraclavicular glands surviving 8 and 11 months, and 2 with pulmonary metastases surviving 7 and 10 months. The 27 cases showing no improvement of the response were all very advanced and included : carcinoma of breast, 4 cases ; carcinoma of ovary, 4 ; carcinoma of mouth, including tongue, 4 ; extrinsic carcinoma of larynx, 1 ; post cricoid carcinoma, 1 case (surviving 4 months) ; carcinoma of antrum, 1 case ; carcinoma of stomach, 1 ; carcinoma of oesophagus, 2 ; carcinoma of colon, 2 ; carcinoma of rectum, 1 ; teratoma testis, 1 ; reticulosarcoma, 2 ; lymphosarcoma, 1 ; squamous carcinoma of skin, 1 ; and melanoma, 1.

Of the 36 cases still living, the 13 showing unexpectedly good palliative response were : carcinoma of the tongue, 4 cases, of which 3 were in the posterior third ; recurrent anaplastic carcinoma of the nasal fossa, 1 ; extrinsic carcinoma of larynx involving the pyriform fossa with fixed secondary glands, 2; post-cricoid carcinoma with pulmonary metastases, 1; carcinoma involving the whole breast with mobile axillary glands, 1; osteosarcoma of ilium, 1; and carcinoma of the skin 3 cases, of whom 2 were of extremely extensive basal cell lesions of the face with gross tissue destruction, which had been radioresistant but appeared to become radiosensitive with the combination of the compound and X-radiation. The 23 cases showing no improvement of the response were: carcinoma of mouth including tongue, 4; secondary glands from carcinoma of lip, 2; intrinsic carcinoma of larynx, 1; carcinoma of oesophagus, 2; inoperable carcinoma of colon, 1; squamous carcinoma of anus, 1; carcinoma of vulva, 1; squamous carcinoma of skin, 1; fungating carcinoma of breast, 1; carcinoma of uterine cervix, stage IV, 2 cases; advanced ovarian carcinoma with ascites, 2 cases; advanced carcinoma of bladder, 1; mediastinal tumour, 1; and cerebral tumour, 3 cases.

Of especial interest are the 2 cases of advanced basal cell carcinoma of the face which were radioresistant after much previous treatment and apparently became radiosensitive again with the combination of the compound and radical X-radiation.

It is important to emphasize that the use of the compound does not prevent the development of metastases, or obviously reduce their incidence.

*Carcinoma of the bronchus.*

The first criterion for the selection of the cases is histological verification of carcinoma of the bronchus either at bronchial biopsy or post-mortem.

The *control* group, treated by X-radiation alone, consists of 13 cases, with the following times of survival in months, to the nearest month, from the beginning of the X-ray treatment: 8, 8, 7, 6, 5, 4, 4, 3, 2, 2, 2, 2, 2. Of these, 4 received radical X-ray therapy, with minimum tumour doses of 5000 r. in 5 weeks, 4500 r. in 25 days, 3250 r. in 23 days, and 3000 r. in 18 days; these cases survived 8, 4, 6, and 8 months respectively. The remaining 9 cases received palliative X-ray therapy, with smaller doses and usually larger fields. The histological verification was at post mortem in 4 cases, which survived 8, 5, 2 and 2 months; the diagnosis in the remaining 9 cases was based on bronchial biopsy. All the cases in the control group are dead.

The *treated* group, which received X-ray therapy and the compound by intramuscular injection, consists of 9 cases, with the following times of survival in months, to the nearest month, after the beginning of treatment: for the 2 cases still living (with symptoms) on 20.ix.48, 12 and 11, and for those dead, 9, 9, 9, 6, 5, 4, 3. All received palliative X-ray therapy except the one case which survived 4 months and had been given a minimum tumour dose of 3000 r. in 20 days. All but one case surviving 9 months were diagnosed on the bronchial biopsy.

Although the groups are small, the results of the combined use of the compound and X-ray therapy show a small improvement in length of survival, which must be regarded as significant, upon the results of X-ray therapy alone. Assuming that the parent distributions from which the control and treated groups are drawn, are not grossly different from normal, as discussed below, it appears valid to apply Student's $t$ test. The absence of systematic error must be assumed.

For the control and treated groups specified above, the mean survival time is 4·23 months for the *control* group and 7·56 months for the *treated* group. The difference between these mean survival times is significant, since for 20 degrees of freedom, $t = 2{\cdot}838$ and $P_F = 0{\cdot}010$.

For the members of the control and treated groups receiving palliative X-ray therapy, the mean survival time is 3·22 months for the controls and 8·00 months for the treated group. The difference between these two sub-groups is very significant ; for 15 degrees of freedom, $t = 3{\cdot}98^3$ and $P_F = 0{\cdot}002$.

The mean survival time in the *control* group, treated by X-ray therapy alone, is in agreement with the results of other workers. Schinz and Zuppinger (1937) reported that for 69 cases, the mean survival time after the beginning of X-ray treatment was 4·4 months ; 16 cases (23 per cent) lived more than 6 months and 4 cases more than 1 year. Dobbie (1944) found that of 111 cases receiving palliative X-ray therapy, 26 per cent lived more than 6 months, and of 48 cases treated by radical X-ray therapy 54 per cent lived more than 6 months.

In our own cases, treated by X-ray therapy alone in 1944–47, inclusion of histologically unverified cases for which the clinical diagnosis was reasonably certain and which survived 2 months or more, gives a group of 47 cases with a mean time of survival after the beginning of treatment of 5·23 months. This group is comparable with the whole control group specified above and has a distribution which is not normal, although the divergence from normality is not very convincing ($P = 0{\cdot}03^1$). Inclusion of all the cases with a reasonably certain diagnosis treated in this department before May, 1947, by X-ray therapy alone gives a group of 79 cases with a mean life of 4·15 months. None of these are still living ; 12 cases lived more than 6 months and only one case lived more than 1 year (13 months).

The consistently bad results obtained in carcinoma of the bronchus after X-ray therapy alone with comparable techniques used in different centres makes it possible to carry out an investigation such as that described with reasonable certainty that the results are not invalidated by systematic error.

The results described showing a small increase in the length of survival after X-ray therapy due to the ancillary use of the compound are in accordance with clinical impressions. The improvement in the palliative effect was striking in 3 of the 9 cases in the treated group. Of these 3 cases, one was a woman of 41 years of age who was extremely ill when admitted, and returned temporarily to almost normal health but died after 9 months ; the other two were men of 59 and 60 years of age, and were still living on 20.xi.48, though with returning symptoms due to recurrence, after 13 and 14 months. The unexpectedly good immediate palliative response in these 3 cases was not obviously correlated with the histology but was associated with an unusually severe skin reaction to the radiation. In this connection it is of interest to note that the rare cases responding anomalously well to X-ray therapy alone often show an unusually severe skin reaction.

The use of the compound did not prevent the development of metastases, including cerebral metastases, which occurred in the one of the 2 female patients in the treated group, who survived 6 months.

It is concluded that intra-muscular administration of the compound produces a small but useful improvement in the palliative results of X-ray therapy in some cases of inoperable carcinoma of the bronchus.

*Intravenous administration.*

Administration of the compound by intravenous injection has been used in 20 cases. Of these, 6 cases were in combination with radical X-ray therapy and included : advanced carcinoma of mouth, 2; carcinoma of bronchus, 2 ; post-cricoid carcinoma, 1 ; and adeno-carcinoma of ethmoid, 1 case. The 4 cases in combination with palliative X-ray therapy were : advanced carcinoma of mouth, 1 ; advanced carcinoma of colon fungating through anterior abdominal wall, 1 ; and carcinoma of ovary, 2. The 10 cases in which the compound has been used alone had all received previous radical X-ray or radium treatment and had recurrences for which in the usual way no further treatment could be done. These cases were : carcinoma of tongue with widespread extension and fixed secondary glands, 2 ; secondary adenocarcinoma in lymph glands of left supra-clavicular region and posterior triangle, with primary in transverse colon, 1 ; carcinoma of bronchus, 2 ; extrinsic carcinoma of larynx with fixed secondary glands, 1 ; fibrosarcoma of neck, 1 ; secondary glands from carcinoma of ear, 1 ; melanoma with hepatic and pulmonary metastases, 1; and generalized reticulo-sarcoma, 1. In at least 4 cases there was subjective improvement including relief of pain. In the 2 cases of advanced squamous carcinoma of the tongue and the case of secondary adenocarcinoma in glands of neck, in addition to the subjective improvement, there was some retrogression of the tumours. This was confirmed histologically in the case of the secondary adenocarcinoma. It is emphasized that these effects are only palliative and require much further investigation.

### SUMMARY.

Clinical trials of large doses of tetra-sodium 2-methyl-1 : 4-naphthohydro-quinone diphosphate, a synthetic vitamin K substitute, mainly in conjunction with palliative X-ray therapy, have been carried out in 116 patients with advanced malignant tumours. The compound is of very low toxicity and is administered by intramuscular and perhaps preferably, intravenous injection. Large intraven-ous doses produce focal pain in the region of the tumour in some cases, especially where there is bone involvement.

A general survey of the use of the compound, administered by intramuscular injection, in combination with X-ray therapy, showed an unexpectedly good palliative response in at least 23 out of 73 patients with various common types of advanced malignant tumours, other than carcinoma of the bronchus.

In a small series of histologically verified cases of inoperable carcinoma of the bronchus, the treated group of 9 cases who received X-ray therapy and the compound by intramuscular injection, showed a mean time of survival after the beginning of X-ray treatment of $7{\cdot}5^6$ months, while the control group of 13 cases, receiving only X-ray therapy, had a mean survival time of $4{\cdot}2^3$ months. For the members of the treated and control groups who received palliative X-ray therapy, the corresponding mean survival times were $8{\cdot}0^0$ months and $3{\cdot}2^2$ months. These differences between the treated and control groups are con-sidered to be significant.

Intravenous administration of the compound has been used in 20 cases. It was used alone in 10 very advanced cases and in 3 of these there was at least subjective improvement.

It is concluded that tetra-sodium 2-methyl-1 : 4-naphthohydroquinone diphosphate in large doses produces a small but useful improvement in the palliative results of X-ray therapy in some cases of advanced cancer.

I am indebted to Dr. F. Wrigley, of Roche Products Ltd., Welwyn Garden City, for arranging supplies of "Synkavit."

### REFERENCES.

AHLSTRÖM, L., EULER, H. V., AND HEVESY, G.—(1944) *K. svenska VetenskAkad. Arkiv Kemi* 19A, No. 9.

BADGER, G. M., ELSON, L. A., HADDOW, A., HEWETT, C. L., AND ROBINSON, A. M.—(1942) *Proc. Roy. Soc.* B., **130**, 255.

DOBBIE, J. L.—(1944) *Brit. J. Radiol.*, **17**, 107.

EULER, H. V., AND HEVESY, G.—(1942) *K. danske vidensk. Selskabs. Biol. med.*, **17**, 8.

*Idem* AND SOLODKOWSKA, W.—(1947) *Ark. Kemi, Min. Geol.*, 24A, No. 24.

FOSTER, R. H. K.—(1940) *Proc. Soc. exp. Biol., N.Y.*, **45**, 412.

FRIEDMANN, E. J., MARRIAN, D. H., AND SIMON-REUSS, I.—(1948) *Brit. J. Pharmacol.* **3**, 263.

FROMHERZ, K.—(1941) *Z. Vitaminforsch.*, **11**, 65 (quoted from Morton, R. A.)—(1942) *Ann. Rev. Biochem.*, **11**, 385.

HEVESY, G.—(1945) *Rev. mod. Phys.*, **17**, 102.

HOLLOMAN, A. L.—(1947) 'Approaches to tumor chemotherapy.' *Amer. Ass. Advancement Science*, Washington, p. 273.

HOLMES, B. E.—(1947) *Brit. J. Radiol.*, **20**, 450.

KALCKAR, H. M.—(1945) *Fed. Proc.*, **4**, 248.

LEHMANN, F. E.—(1942) *Verh. Ver. Schweiz. Physiol.*

*Idem*, LÜSCHER, M., AND HUBER, W.—(1945*a*) *Revue suisse Zool.*, **52**, 342.—(1945*b*) *Ibid.*, **52**, 249.—(1945*c*) *Ibid.*, **52**, 354.

MEIER, R., AND ALLGÖWER, M.—(1945) *Experentia*, **1**, 57.

MITCHELL, J. S.—(1942*a*) *Brit. J. exp. Path.*, **23**, 285.—(1942*b*) *Ibid.*, **23**, 296.—(1942*c*) *Ibid.*, **23**, 309.—(1943) *Brit. J. Radiol.*, **16**, 339.—(1944) *Rep. Brit. Emp. Cancer Campgn.*, 62.—(1946) *Schweiz. med. Wschr.*, **76**, 883.

*Idem* AND SIMON-REUSS, I.—(1947) *Nature*, **160**, 98.

MOLITOR, H., AND ROBINSON, H. J.—(1940) *Proc. Soc. exp. Biol., N.Y.*, **43**, 125.

SCHINZ, H. R., AND ZUPPINGER, A.—(1937) *Siebzehn Jahre Strahlentherapie der Krebse.* Leipzig (Thieme), p. 255.

SHEAR, M. J.—(1947) 'Approaches to tumor chemotherapy.' *Amer. Ass. Advancement Science*, Washington, p. 236.

SMITH, J. J., IVY, A. C., AND FOSTER, R. H. K.—(1943) *J. Lab. clin. Med.*, **28**, 1667.

STOWELL, R. E.—(1945) *Cancer Res.*, **5**, 169.